# Interhemispheric Acute Subdural Hematomas

**Published:** 2011-04-01

**Authors:** N N Kawoosa, A R Bhat, B Rashid

**Affiliations:** 1 Department of Accident and Emergency, Sher-i-Kashmir Institute of Medical Sciences (SKIMS), Soura, Srinagar, Kashmir, 190011, INDIA; 2 Department of Neurosurgery, Sher-i-Kashmir Institute of Medical Sciences (SKIMS), Soura, Srinagar, Kashmir, 190011, INDIA

**Keywords:** Acute subdural haematoma, ASDH, Interhemispheric, Management

Dear Editor,

Acute subdural hematoma (ASDH) is a neurosurgical emergency. Interhemispheric acute subdural hematomas have been reported infrequently in the past. They are usually considered to be a distinct entity because of their unusual location and the fact that their management is still a matter of debate. The most common sites for ASDH are the fronto-parietal convexities and the middle cranial fossa. ASDH rarely presents in the interhemispheric fissure. Interhemispheric ASDHs were first described by Aring and Evans[1] in 1940 and only about 100 cases have been reported till 1997.[2][3][4] Interhemispheric ASDHs are usually unilateral but bilateral hematomas have also been reported. Interhemispheric ASDH usually occurs posteriorly in the majority of cases. They usually occur in patients with bleeding disorders and are associated with trauma in 83% of cases.[2] Other causes include history of birth trauma, forceps delivery, child abuse with shaking, hemodialysis, anticoagulation and aneurysmal bleeding.[5] Even with the increasing number of motor vehicle accidents resulting in severe head injuries, it is surprising that the diagnosis of interhemispheric ASDH has not been made more frequently. The natural history of interhemispheric ASDH is not fully understood because of the early surgical intervention. The amount and the direction of force that can produce an interhemispheric ASDH remain controversial. The hematoma occurs from bridging veins between the parietooccipital cortex and superior sagittal sinus or between the falx cerebri and the medial surface of the cerebral hemispheres. Fruin et al.[6] suggested that an occipital blow in the sagittal plane lead to an interhemispheric ASDH because of the anatomic orientation of the veins in the interhemispheric fissure, which tend to course antero-medially from the cortex to the midline sinuses. Before the CT era, it was difficult to detect an interhemispheric ASDH. Though removal of the blood has proved to be an option in the management of these patients, there is danger due to the close proximity of the superior sagittal sinus and bridging veins. Some of these hematomas migrate superiorly (to a more favorable position) with time, as they liquefy. It is also conceivable that if a patient with an interhemispheric ASDH is relatively asymptomatic, initial conservative management might be followed by migration of the clot to a position over the convexity where removal is considerably less dangerous. Thus there is no consensus on the ideal management of these rare hematomas, conservative treatment may be followed in those who are neurologically stable or have concurrent risk factors, while surgical treatment should be reserved for those who have pronounced symptoms or neurological deficits.

**Fig. 1:**
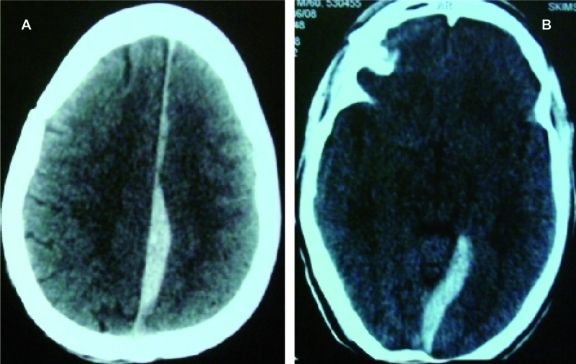
Axial CT scan after head injury following road traffic accident revealing interhemispheric acute subdural hematoma.

In Figure 1: Axial CT scan performed after head injury following road traffic accident revealing interhemispheric acute subdural hematoma. The patient presented with a falx syndrome of contralateral hemiparesis, most marked in the lower extremity.
